# Accounting for One Health: Insights from the social sciences

**DOI:** 10.1051/parasite/2020056

**Published:** 2020-11-03

**Authors:** Jérôme Michalon

**Affiliations:** UMR Triangle – ENS de Lyon site Descartes, Bat D4 (recherche) – 2ème étage 15 parvis René Descartes 69342 Lyon cedex 07 – France

**Keywords:** One Health, Political Sociology, Science studies, Watchword

## Abstract

This paper discusses the relationship between One Health (OH) and the social sciences. Using a comparison between three narratives of the history of OH, it is argued that OH can be studied as a social phenomenon. The narrative of OH by its promoters (folk narratives) emphasizes two dimensions: OH as a renewal of veterinary medicine and OH as an institutional response to global health crises. Narratives from empirical social science work explore similar dimensions, but make them more complex. For political sociology, OH is the result of negotiations between the three international organisations (WHO, OIE and FAO), in a context of a global health crisis, which led to the reconfiguration of their respective mandates and scope of action: OH is a *response to an institutional crisis*. For the sociology of science, OH testifies to the evolution of the profession and veterinary science, enabling it to position itself as a promoter of interdisciplinarity, in a context of convergence between research and policy. In the Discussion section, I propose an approach to OH as an “epistemic watchword”: a concept whose objective is to make several actors work together (watchword), in a particular direction, that of the production of knowledge (epistemic).

## Abstract (fr)

Cet article aborde les rapports entre One Health (OH - « une santé » en français) et les sciences sociales. L’idée que OH peut être étudié comme un phénomène social est défendue, au moyen d’une comparaison entre trois narrations de l’histoire de OH. La narration de OH par ses promoteurs (narrations indigènes) insiste sur deux dimensions : OH comme renouveau de la médecine vétérinaire et OH comme réponse institutionnelle à des crises sanitaires. Les narrations issues de travaux empiriques en sciences sociales explorent des dimensions similaires, mais les rendent plus complexes. Pour la sociologie politique, OH est le résultat d’une négociation entre les trois organisations internationales (OMS, OIE et FAO), dans un contexte de crise sanitaire globale, ayant amené à reconfigurer leurs mandats et leurs périmètres d’action respectifs : OH est *une réponse à une crise institutionnelle*. Pour la sociologie des sciences, OH témoigne des évolutions de la profession et de la science vétérinaire, permettant à celle-ci de se placer en position de promotrice de l’interdisciplinarité, dans un contexte de rapprochement entre recherche et action publique. Dans la partie « discussion », je proposerai d’aborder OH comme un « mot d’ordre épistémique » : un concept dont l’objectif est de faire travailler plusieurs acteurs ensemble (mot d’ordre), dans un sens particulier, celui de la production de savoirs (épistémique).

## Introduction: One Health and social sciences

The purpose of this article is to contribute to reflections on ways to articulate One Health (OH) and social sciences. By “social science”, I mean the disciplinary corpus that has developed since the middle of the 19th century, and which deals with the scientific understanding of human behavior, in its relation to institutions. I include in these disciplines: economics, sociology, anthropology, social geography, history, political science and psychology. Unlike philosophy, the social sciences mobilize empirical approaches to answer the questions they ask themselves. In this respect, they can claim the title of “sciences”. Being themselves social entities, these disciplines and their contours evolve according to periods and geo-political and institutional contexts. Some social sciences may, for example, approach the life science’s epistemology, using laboratory experimental protocols, such as behavioral psychology. Conversely, some disciplines of life sciences use methods that are sometimes close to those of the social sciences, such as “field” ethology, for example. But the core of the social sciences can be recognized by its attachment to the ideas of the historicity of human practices, symbolic relationships to others and to the environment, and the construction of institutions and norms that organize collective life. It is this understanding, this vision of the social sciences, that will be discussed in this article, in particular because it is the one most found in the work on OH.

There are two ways in the literature to consider the relationship between OH and social sciences. The first is to take part in the achievement of the One Health agenda [40]. The aim here is to present the OH agenda as an opportunity for social sciences: opportunity to promote their skills in producing a general understanding of human behaviour: since changing individual and collective practices is at the heart of the OH agenda, whose disciplines would be better qualified than social sciences to objectify the social, political, economic and legal processes that can promote or hinder the management of global and trans-specific health? In addition to this general knowledge of what humans do, there is also knowledge of the local contexts in which the practices to be changed are observed: How can we ensure that the OH agenda can be achieved by taking into account practices, knowledge and representations relating to human health, animal health and ecosystem care [64]? On this topic, the expertise of social sciences is presented as crucial (especially that of anthropology, and in particular development anthropology), for the successful deployment of global health, which would not result in the imposition or plating of exogenous norms and practices (by Northern countries) on heterogeneous cultural realities (symbolic and material). The model of “social acceptability” (in its most asymmetrical form) or “co-construction” (in its more egalitarian version) then appears. The role of social sciences would be to ensure compatibility between public policies, technological innovations or development assistance mechanisms and a “target” population that is supposed to receive, accept or co-construct them. In this respect, the OH agenda presents a very favourable ground for the application of survey methods and co-construction mechanisms, mastered by social sciences and having already proved their worth on other topics [13]. Social sciences therefore propose a dual service offer to the OH agenda: a *cognitive* offer, highlighting knowledge of the diversity in practices and representations of human, animal and environmental health; and an *operational* offer, highlighting knowledge of the processes by which it is possible to arrange this diversity in such a way that it serves the OH agenda. This operational proposal also applies to the coordination of the actors (politicians, scientists, NGOs) who are supposed to work together within the framework of OH. Here again, social sciences are volunteering to improve the understanding of different institutional and/or disciplinary cultures and to go beyond the logic of “silos” [45]. Generally formulated in purely epistemological terms, the opportunity for social sciences to be included in the OH agenda is not, however, disconnected from a context of institutional marginality: OH represents a significant call for financially and epistemologically “dominated” disciplines, in the academic world. The possibility of linking up with multidisciplinary programmes, firmly funded and politically supported at the international level, is not very frequent for social sciences, which might explain their willingness to take an active part in the OH agenda.

This positioning, this service offer, is not the only way social sciences can grasp OH. A second option is to analyse OH as an object, as a social phenomenon in itself. This consists in understanding the context in which “One Health” has emerged, which actors promote it, who makes it emerge, and in which networks it circulates, and how it is appropriated by some according to their interests, their strategies, etc. This approach has already proved its worth, and has made it possible to understand the emergence of OH in the wake of changes in public health management. A large body of literature has focused on OH as the ultimate avatar of the globalisation mechanisms of human health [26], and animal health [18]. In this trend, a significant number of studies present OH as a symptom of a new way of managing health risks, making animal health control an opportunity to impose “biosecurity” [20] on a global scale [16, 17, 40]. This descriptive and analytical work shows that the emergence of OH is linked to varied and complex issues, which are partly beyond the reach of the promoters of the OH agenda. An overview of OH, its history and ramifications seems difficult to obtain, and the interest of social science work does not lie in the ambition to produce this “meta”, “totalizing” point of view on OH. On the contrary, social sciences can be useful to draw attention to some (not all) dimensions that can be obscured by those who think and implement the OH agenda, notably because of the professional and disciplinary interests that the OH agenda could help them serve. What does OH stand for? Who is talking *about* OH? Who speaks *in the name of* OH? Who speaks *for* OH? What does OH do? What does OH make actors do? Why OH now? These dimensions can be quite basic from the point of view of social sciences, but they can be neglected by OH promoters themselves, even (and perhaps even more) when they intend to report them, and give them meaning. This article aims to show that a social science perspective can enrich the understanding of the emergence of OH, and enrich the point of view of the actors involved in the promotion of OH.

To demonstrate this, I will present three types of narratives of the emergence of OH. First, I will focus on narratives produced by authors explicitly engaged in the OH agenda (folk narratives), to highlight how they explain “why OH now”? Then, I will present a narrative produced from a political sociology perspective, then another produced from a sociology of science perspective. I will show that these narratives are not necessarily disconnected from each other: although the narratives of the social sciences differ from folk narratives, they take up certain elements of them, providing them with a new perspective.

In the discussion part, I will propose a way to continue the sociological exploration of OH. Noting that the formal dimension of OH has not yet been taken sufficiently into account to qualify what OH is, I will propose the notion of “epistemic watchword”.

## Materials and methods

This article is based on the reading and analysis of more than 20 articles, both in social sciences and life sciences. Their common point is to propose, in varying proportions, narratives of the history of OH. Two articles are presented in more detail. They were chosen because there is still little empirical research in the social sciences that traces the origin of OH. They seemed to me to be the most advanced in the application of a sociological approach to the object OH. Moreover, they present two different aspects of this approach: one is based on a qualitative survey, mobilizing interviews with actors who contributed to the fabrication of the OH concept. The other is based on an in-depth bibliometric survey, which gives access to the evolution of the scientific meanings of the OH concept. In both cases, an empirical, evidence-based approach is applied. The choice of these papers was therefore not made on the basis of their impact in the field of social sciences interested in OH. Rather, the aim is to present two archetypal models of social science research. Moreover, these two research styles correspond here to two sub-fields of the social sciences: political sociology and sociology of science. The first documents the power relations that exist within and between actors and institutions related to public decision making and politics in general. The second analyzes the social dynamics within and between institutions and actors in the scientific world, and approaches the production of knowledge as a social phenomenon in its own right. Insofar as OH presents itself as a new way of producing knowledge and governing public health policies, these two articles provide a highly complementary perspective.

This text is more of an essay than a rigorous scientific analysis or a systematic review. Above all, it seeks to identify avenues for reflection on the sociological understanding of OH.

## Results

### New bottle or new paradigm? The folk narratives of OH

Talking about “folk narratives” is a way of indicating that OH does not develop independently of the stories that are made by the promoters of its genesis. These stories are woven from factual and speculative elements, seeking to account for the emergence of OH and to give particular guidance to the application of the accompanying agenda. The common point of these stories is that they are produced by authors working in close proximity to the OH agenda, in finalised research, sometimes within international organisations that promote it. This is what qualifies these stories as indigenous or “folk” and gives their content a particular orientation, both *descriptive* and *strategic*.

These narratives thus oscillate between two ways of presenting OH: either as the new version of a traditional vision of health, which would have been rediscovered; or as a radically new paradigm, appearing in new circumstances, which required innovative responses.

In the first narrative, OH’s vision of trans-specific health appears not to be new. Obviously, health care would have at all times required thinking about the interactions between humans, animals and ecosystems. Some authors explain that: “*the word HEALTH itself can be interpreted as an acronym composed of: Humans - Ecosystems - Animals - Living - Together - Harmoniously.*” ([14], p. 415). This holistic and systemic dimension may have been forgotten, along the development of scientific human medicine. Only veterinary medicine may have preserved the memory and wisdom of this “global” thought. OH philosophy is said to be at the very heart of veterinary science and practice. OH could only be “old wine in a new bottle”; the novelty of the thing residing only in the official recognition of the importance of this philosophy carried since always by veterinarians [27, 52]. From such a perspective, making the history of OH is making the history of veterinarians, and presenting some of them as precursors of OH. Although this narrative is rich in factual elements, the way they are arranged clearly aims at enhancing the value of the veterinary profession, in an exercise of legitimization well analysed by historians [31, 58] and sociologists [6]. Since its creation, the veterinary profession has legitimized itself by emphasizing its indirect contribution to the preservation of human health. In view of the multiplication of narratives discussed here, it can be assumed that OH provides a new opportunity for the profession to mobilize this rhetoric of interconnection of health processes and centrality of veterinary expertise, to account for and take charge of them. Especially since, as we will see later, veterinarians have already tried to propose integrative concepts, allowing the link between animal and human health (or illness), such as “One Medicine” or even the concept of “zoonosis”.

The second folk narrative of OH’s genesis is found quite regularly in the Introduction section of articles wishing to implement the OH approach or presenting results from its application. Unlike the first version, this one insists on the radical novelty that OH represents. Its appearance can be traced back to the mid-2000s, following a series of global health crises that began in the late 1990s. SARS, the H1N1 virus, but especially the H5N1 virus, are presented as major events that have shown the organizational and scientific limitations of the systems of actors in charge of global health management. These emerging diseases, which have fallen under the control of health authorities and international organisations, seem to have overwhelmed their prediction, modelling and coordination capacities. This is what would have pushed these actors to adopt a systemic, holistic framework of thought to deal with the emergence of new health risks. The chronology found in this type of narrative includes the Manhattan Principles (2004), under the aegis of the Wildlife Conservation Society, and their adoption by the American Veterinary Medical Association (AVMA) following the launch of the One Health initiative in 2008, then by WHO, FAO and OIE between 2008 and 2010 (concept note on One World One Health – reiterated in 2017), and by the US Centers for Disease Control and Prevention. In this OH narrative, a whole series of institutional responses to events requiring a new type of governance and expertise is listed. This “rhetoric of response” is best illustrated by the article by Scoones and Forster [54], which proposes an extensive double entry table in the Appendix, showing in the left column the biological events (disease biology) that occurred between 1997 and 2007, and in the right column the institutional responses (policy responses). As unexpected as pandemics, OH then seems to emerge as a set of new answers to questions that are no less unexpected. This form of narrative, mobilizing a chronological and cumulative perspective (through the succession of strong statements and actions from powerful actors), places OH in a certain relationship to temporality: OH is a *project* that is continually being constituted, each new stage being the opportunity to list new obstacles to its achievement and new challenges to be met. The notion of “project” has been well analysed by sociologists, mostly as a principle of governance and a new tool for managing and controlling behavior [5]. In this narrative, OH then becomes *a horizon that encourages action*, an aspect to which I will return later. In addition to the chronological dimension, the mention of certain powerful actors who have adopted OH (international organisations, certain NGOs and certain professional organisations from powerful States) also gives this narrative an incentive function. Associating OH with these particular actors (and not others) gives the concept an authority and power to convince anyone of its interest in integrating into the OH agenda.

OH’s folk narratives, whether they insist on the radical novelty of the concept or on its inclusion in the continuity of past practices, have in common to serve the interests of their authors. Such a conclusion will probably come as no surprise to anyone in the world of social sciences. Historians have taught us: writing history is anything but a trivial act. For a long time reserved for the elites, writing history was a tool of power and legitimization of power. OH’s stories are no exception. It is therefore interesting to find other narratives, produced by authors whose proximity to the OH agenda is less significant, who approach the question of power relations between its promoters in a more explicit way.

### A response to a governance crisis: OH as an object for political sociology

After these stories where OH appears as an institutional response to events requiring a new form of governance and expertise, let us now turn to a narrative resulting from research in political sociology; in this case, the work of the American sociologist Yu-Ju Chien, who published a paper entitled “*How did international agencies perceive the avian influenza problem? The adoption and manufacture of the ‘One World, One Health’ framework”*. [12] This research consisted in qualitative research, investigating three international organisations (WHO, FAO and OIE), involving interviews with officials of these organisations and also work on archives and grey literature. Chien explains the context in which the OH concept, despite its vague nature, has been adopted. The health crises of the early 2000s, already mentioned, have indeed generated tension between the three organisations. But unlike the folk narratives, insisting on the organisational limits that health crises brought to light, Chien refers rather to a crisis of legitimacy of the three organisations, in the making for a long time. The spread of H5N1 has led to conflicts over how to limit its impact. The preventive slaughter of poultry to limit contamination is a point of tension between WHO, which advocates the solution as a public health measure, and FAO and OIE, which have in mind the consequences of the measure on livestock farmers and animals. Conflicts of mandate appear as follows: protecting human health (WHO) and preserving animals and economic interests related to animal health (FAO and OIE) contradict each other. It can be added that tensions arise between the OIE and the FAO precisely because each claims expertise and action on animal health protection, with slightly different perspectives (public health for the OIE/livestock and development support for the FAO). On the relationship between the OIE and FAO regarding “animal health” and “zoonosis”, see Camille Torres’s dissertation [60] and Frédéric Vagneron’s work [61]. These inherent tensions in the mandate of these international organisations are exacerbated by a competition for access to the exceptional funds released by some States to respond to H5N1. For example, Canada played a key role in the creation of several coordination bodies between FAO and OIE [60]. In addition to these tensions *between* international organisations, there are also tensions *within* these organisations. Between 2003 and 2008, debates emerged on how best to manage public health problems on a global scale, Chien explains. Three “frameworks of public action” are in conflict, indexed to the different types of expertise found in these international organisations (biologists, doctors, veterinarians, economists, epidemiologists, anthropologists, etc.). Biomedical or technical framing – which could also be called “technocratic” – consists in defending the application of sanitary measures, coming from institutions (in a top-down logic), and whose legitimacy lies in the recognition of their effectiveness in eliminating viruses and limiting their spread. This is a quasi hunting logic, illustrated by a quote from the first consensus note published by the three organisations: *“‘Find it fast - kill it fast - stop it spreading’ (FAO 2008: 13)”* cited by Chien ([12], p 217). This biomedical framework is promoted mainly by biomedical experts (human and veterinary medicine). Rather supported by economists and social science researchers, the “societal” or “democratic” framework defends that health problems must be managed collectively, in a bottom-up logic, with the people directly concerned, taking into account the diversity of social contexts and cultural representations, which will make them all the more effective. It is as much a question of ensuring the democratic legitimacy of the measures adopted (an essential element for talking about public policy) as it is of improving their implementation, from a strategic perspective. We find here the elements mentioned in the Introduction, about the positioning of social sciences in relation to OH. Finally, the “environmental” framework emphasizes the need to think about the ecosystemic impacts of ways of managing public health problems, and to always keep in mind the sustainability of the proposed solutions. Led by conservation biology experts, this framework also emphasizes the instrumental dimension of ecosystem concern: depending on whether or not ecosystem functioning is taken into account, the spread of pathogens can be accelerated or curbed. Even though these three frameworks do not have the same institutional weight (biomedical framing is dominant, and the other two are more marginal), they were subject to discussion between 2003 and 2005, and do not help to ensure that the three international organisations present a united front against the challenges of global health and health crisis. The dynamic of collaboration between organisations, supported by some of their Member States, will consist in reducing conflicts between these different frameworks, while respecting the values and expertise that each represents. The publication of the tripartite concept note in 2010 on One World One Health is the result of this effort. OH emerges here as a new, integrative framework. Its strength lies in the fact that it has managed to reconcile the values of each of the three competing frameworks (modernity – equality – sustainability), and to call for the creation of new knowledge, merging or linking hitherto fragmented areas of expertise. Here, the adoption of OH is no longer an institutional response to health crises, but more precisely, it is a *response to institutional crises* generated or revealed by these health crises, in terms of governance and expertise. OH is thus described by Chien as a tool for pacifying relations between and within international organisations. The success of the operation is based on the “blurry”, “vague”, “imprecise” nature of the OH concept, which allows each actor to reformulate its interests, legitimacy and skills as best it can. For each of the actors, OH provides *symbolic* legitimacy since it accentuates the *common points* between the missions of the actors while *attenuating* their differences: a single health is the objective that all must serve. And OH provides *operational* legitimacy since it accentuates the differences between the skills and expertise of the actors and reduces their common points: varied and complementary expertise is necessary to achieve the objective. The OH agenda will be constituted as the horizon by which these two contradictory dynamics will be articulated. In short, OH is a tool for pacification because it provides international organisations with a common agenda and recognises that they have complementary skills to carry it out. OH is therefore approached here as a tool of governance, eminently political. However, a purely institutional reading is not enough. While OH calls for collaboration between different actors in public health policy, it also encourages the production of a new and innovative form of knowledge. Another narrative of OH, just as relevant as the one just described, would then be to look at the cross-fertilisation of the scientific disciplines invited to work together.

### A symptom of science/policy convergence: OH as an object for sociology of science

This third narrative is proposed by Angela Cassidy, a sociologist who worked with British historian Abigail Woods as part of a broad research programme to establish the history of OH in the context of developments in modern medical science [65]. Some of the results of this programme are presented in a chapter of a collective book dedicated to the empirical and critical analysis of interdisciplinary research [11]. This bibliometric and historical study covering the period 1970–2014, leads to a narrative in which OH appears to be the result of long and fluctuating interactions between various scientific disciplines, in particular veterinary medicine, between different ways of conceiving science (epistemic models), and between the academic world and international organisations (Fig. 2).

**Figure 1:**
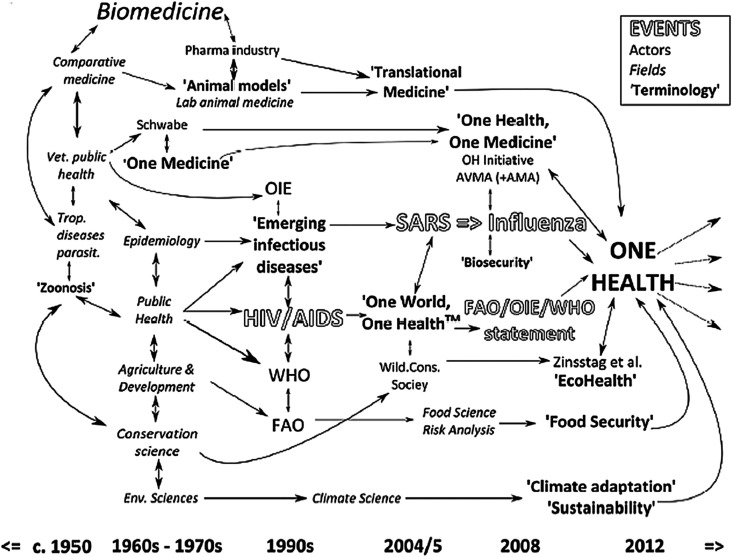
Overview of the One Health history – Cassidy (2016) – p. 226.

One of the most interesting results is a synoptic representation of OH recent history (Fig. 1). It includes both the “events” already mentioned (health crises and statements by international organisations), but also institutional and scientific “actors”, “research fields”, and, above all, the terminologies that preceded or inspired OH. The relationships between these different “items” are also represented. This scheme is very telling, and reflects the complexity of what led to the adoption of OH as a key word recognised by both institutional actors and scientific disciplines. Far from OH’s unique genealogies, it can be seen that multiple attempts to promote OH’s integrative philosophy have been made since the 1950s, resulting in various conceptual and semantic innovations. The conclusions that can be drawn from this perspective are many. But at the risk of simplifying Cassidy’s remarks, I propose to highlight one of them, which is particularly salient. The appearance of OH in the mid-2000s results from the encounter between two previously developed ways of thinking jointly about human and animal health, corresponding to two terminologies: “One Medicine” and “One World One Health”. The One Medicine concept, attached to the name of veterinarian and epidemiologist Calvin Schwabe, is part of the continuity of an old discipline – comparative medicine – and the development of veterinary public health, while maintaining privileged links with research on animal models and translational medicine. In a nutshell, One Medicine gathers actors initially located on the “academic” side and interested in “pure” research questions, without favouring one disease over another: the link between human and animal health represents an *epistemic* challenge. The concept One World, One Health (OWOH) appears at the crossroads of the field of international relations, in which the concept One World has been used since the 1950s – and the fields of public health and epidemiology, as practiced precisely in international organisations. As already mentioned, management of infectious diseases, from a public policy perspective, is at the heart of the adoption of OWOH. It is important to remember that OWOH is first and foremost a term coined by the NGO Wildlife Society, which will register the copyright. Between 2004 and 2010, OWOH has been used and discussed by international agencies, which finally transformed it into One Health, for copyright reasons. It is therefore in the world of action (NGO’s and international organisations) that OWOH has mainly circulated. Simplifying again, OWOH is more in line with an applied science, or “finalised” in the service of public health. Here, the link between human and animal health is first and foremost a public policy issue. In short, OH is the meeting point between two ways of seeing and practising veterinary science, one more academic, oriented towards research and the other more political or institutional, oriented towards action in general and public action in particular. These are, therefore, also two ways of conceiving the production of knowledge that meet with OH, whose advent also testifies to an intensification of the links between science and public action [28, 34], also in the making for several years.

**Figure 2:**
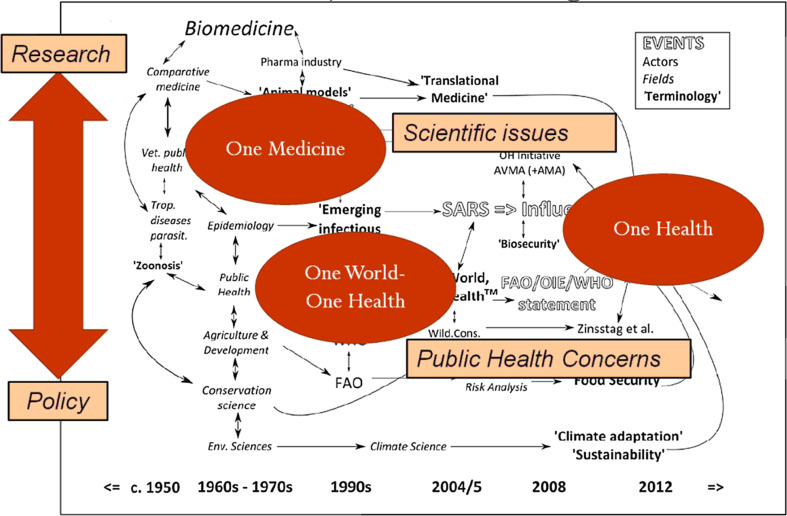
One Health at the meeting point of research and policy – Author – Adapted from Cassidy (2016).

Indeed, since the 1990s, some sociologists have been talking about a global change in the way they think about the production of scientific knowledge [23, 48]. For a long time, they have argued that the scientific world was empowered vis-à-vis the rest of the social world, and it was able to decide for itself how science should be done, its orientations and its institutional organisation. Described as “mode 1” of knowledge production, this situation is gradually being challenged, both for institutional, financial and ideological reasons, considering that science must serve society, and not the other way around. We would thus have switched to “mode 2” of knowledge production, characterised by bringing together the scientific world and extra-scientific actors (industrialists, NGOs, public action), promoting applied and useful research, “context driven”, “problem-focused”, co-constructed with citizens, interdisciplinary, transdisciplinary or post-disciplinary. Part of the research in sociology of science also points to the need to rethink the division between the natural sciences and the social sciences [33, 41], which is arbitrarily constructed according to a disciplinary logic (instead of an object-centered logic). For these sociologists, it is indispensable to reconfigure the relationships between disciplines starting from the objects, and not the other way around. These works thus describe another way of conceiving the production of knowledge, which fits very well with the one promoted by OH: interdisciplinary and centered on one object (health) supposedly able to overcome the barriers between natural and social sciences. For a critical synthesis of this work, see Shinn [55]. It is then very clear that OH corresponds to this new model of knowledge production, which is widely supported by scientific and academic institutions. The veterinary profession has been able to seize the opportunity of such a context, through the promotion of OH. Cassidy notes the strong investment of the veterinary world in OH, and notes the recurring calls from its stakeholders for interdisciplinary, collaborative research to be produced. Nevertheless, if we look at which publication media have received the most OH-stamped research, the weight of the veterinary discipline is overwhelming: 61% “One Health” labelled publications are published in veterinary journals. Cassidy even points out that compared to previous terminologies (One Medicine, Comparative medicine, One World One Health), the weight of veterinary medicine has *increased* in the production of knowledge from an OH perspective. Finally, the adoption of OH has positioned veterinary medicine as a leader in interdisciplinary research on public health issues. Thus, the analysis leads us to understand how the promotion of interdisciplinary research can, paradoxically, strengthen the institutional weight of certain disciplines [32].

If OH’s narrative from a political sociology perspective makes it the product of an institutional *crisis*, Cassidy’s narrative from the sociology of science insists on an institutional *transformation*: (1) the unprecedented rapprochement between research and policy, scientific institutions and international agencies [22], of which the success of the notion of “evidence based policy” is a sign [42], involving a redistribution of knowledge production capacities (research policies no longer depending solely on the academic world), (2) but also the increased promotion of interdisciplinarity, also leading to a weakening of the weight of certain disciplines, to the benefit of others which present themselves as interdisciplinary by *nature*.

While the narratives presented here take up some elements of OH – OH’s folk narratives as a renewal of veterinary medicine, and/or OH as an innovation of international public action – the added value of an empirical approach in the social sciences is to escape from a form of reductionism that would consist in having to choose an official version of OH’s history. The emergence of OH is addressed by the social sciences at different scales, behind the scenes of the functioning of international organisations, or in the long term as the institutional contexts in which science and public action are conceived and practiced evolve. It is a whole social context that is depicted, as well as the collective actors who have worked to make OH a powerful concept, in relation to which a certain number of actors are invited to position themselves. Thus, rather than directly serving the interests of those who write them (and thus giving them power), these narratives, on the contrary, make it possible to better understand where OH’s attraction comes from: this power of OH is explained by the power of the actors who promote it collectively.

## Discussion: OH as an epistemic watchword

As demonstrated above, the retrospective sociological approaches of the manufacture of OH are crucial to understand what OH stands for. However, little has been written so far about *the right way to name* what OH is. Again, there are numerous folk terminologies: “Notion”, “Concept”, “Agenda”, “Approach”, “Paradigm”, “Slogan”, “Umbrella Term”. These concepts are used without justification by the authors, and without the consequences really being drawn from this use. Talking about an agenda or about an approach certainly does not have the same symbolic weight. Here too, a social science approach can be mobilised to help find an appropriate qualifier, adapted to the sociological reality of what OH is. Let’s look at what terms have been proposed by social scientists to describe OH and others similar concepts.

Chien [12] speaks of OH as a “framework”, thus using the terminology of international agencies, but which clearly reflects the non-binding nature of what OH is, because some of these organisations have limited normative power over the policies of their Member States, and which, on many subjects, must be content to guide the eyes of national decision-makers, to *frame* them. For example, on FAO’s difficulty in enforcing animal health crisis management principles, beyond just “framing”, see Torres [60]. In addition, framework refers to the need for coordination between these organisations, which also implies a certain latitude, which is well reflected in the term “framework”. The notion therefore covers the function that OH promoters *intend* to give it. Nevertheless, at the end of the investigation, Chien prefers the notion of “boundary-object”, because it better reflects what OH’s vagueness produces: because OH is not clearly defined, each actor can project what they want, and appropriate it all the more easily, and to reaffirm the specificity and complementarity of their expertise. In a word, talking about “boundary-object” better reflects what actors *do* with OH among actors: working together and, in the same gesture, reaffirming what makes them different.

Nevertheless, OH is not an object, it is a word. This specificity seems too important not to be diluted in the vocabulary of the boundary-object, inherited from *science studies*, which can cover a wide variety of things: technical objects, scientific fields, even institutions [56]. OH is a word and perhaps there is more to explore about that. As Chien suggests, the *formal* dimension probably contributed to the adoption of OH by international organisations: *“Most officials at the three agencies recognised that the OWOH slogan is catchy and appropriate.”* ([12], p. 219) Precisely, the principles OH covers have been appropriate because OH is a word and a “good word”. What concept should we use to talk about OH, how should we name it taking into account this formal specificity?

Bernadette Bensaude-Vincent [4] proposes the term “buzzwords” to designate concepts such as sustainable development, responsible innovation, personalised medicine, and green technology for example. Like Chien, Bensaude-Vincent indicates that conceptual vagueness plays an important role in the coordination power of these concepts: “*As shallow linguistic units deprived of substantial meanings, they create a ‘trading zone’ that allows different stakeholders to communicate*.” ([4], p. 250). But for Bensaude-Vincent, this conceptual blur is due to the very shape of the buzzword. Like advertising slogans, these buzzwords take their form from marketing and the business world. They are designed to be easily identifiable, distinctive and memorable: catchy. According to Bensaude-Vincent, the multiplication of these buzzwords in the world of technosciences is as much a sign of the import of economic thought into scientific activity as it is a sign of a change in the scientific world, characterised by an increasing number of scientific institutions, increased internationalisation and competition, as well as a need for coordination to face this competition. In a word: buzzwords are born from the economisation of the scientific universe. From Bensaude-Vincent’s analysis, I would like to retain these two dimensions: (1) the indexation of conceptual vagueness on the necessarily synthetic form of buzzwords, and (2) the fact that their purpose is to influence the production of scientific knowledge. These two dimensions plead to approach OH as a buzzword.

Buzzword has an intuitively depreciative or at least critical connotation: buzzword is associated with impermanence, with something superficial, ephemeral, trendy – a connotation that denies OH the possibility of constituting a stable political and scientific project. The term “buzz” also suggests that One Health only generates a form of futile agitation, noise. The epistemic dimension would be denied here: the knowledge produced by OH would, again, only be superficial. For these reasons, talking about OH as a buzzword is not totally satisfactory. I would like to propose another terminology, another concept: that of “watchword”. This notion, which is rather used in the military or political, trade union or activist fields, reflects the fact that OH is an injunction to *collective* action, conceived as such, which other terms do not say or in a less obvious way. The notion of watchword explicitly emphasizes OH’s strategic and operational dimension: the link with public policies becomes clearer [7]. Moreover, the formal dimension is not neglected here. A watchword is above all a word: like the slogan or the buzzword, the watchword has a particular, synthetic form, which ensures a greater capacity for action.

Indeed, one could simply say that OH is a watchword because it incites a certain number of actors to manage health problems in a different way. I would like to emphasize another type of action to which OH encourages: in addition to coordinating actors to work together, it is about *producing expert knowledge* on the links between human health, animal health and ecosystem conservation. With OH, we are dealing with a particular watchword, a watchword that could be described as *epistemic*, since it invites several actors to engage in the production of knowledge and reflection. OH aims at making scientists work together, which justifies calling it an “epistemic watchword”. Talking about an epistemic watchword thus makes it possible to account for OH’s scientific ancestry, but it also opens up new avenues of research about the current dynamics in the scientific world. While Cassidy’s work clearly shows that OH is the *product* of the evolution of several scientific disciplines, there is still a need to explore the *effects* of OH on other disciplines, and overall on ways of doing science. A first indication of these effects: in a few years, OH has become a “keyword” of research, used to identify work mobilising integrative, interdisciplinary and holistic approaches to health [15]. From “watchword” to “keyword”, the formal dimension of OH is, again, something worth exploring to account for its power.

There is another reason why I prefer epistemic watchword to buzzword. When we look at how actors engage in the reflection about OH, one of the first tasks they are working on is to clarify the definition of OH. This is the same observation that Chien makes when he describes several conferences dedicated to OH, which begin with the participants’ recognition of the extremely vague nature of the concept, and which invariably end with the admission of a failure to develop a more precise definition. “*The final consensus of the meeting was that One World, One Health could mean whatever people want to.*” *(Official OIE – interview 2009)* ([12], p. 220). In a word, what OH makes actors do is thinking about what OH is. We find a rather similar phenomenon in the literature, where several authors question the purely rhetorical or purely semantic dimension of OH: is OH something more than a word, a label, an idea? Countless papers provide examples of how the OH principles have been applied [15], with the aim of proving that OH is not just a word, and that it is a tangible reality. Other articles critically explore the neglected zones [50] and limits of the OH agenda [57]. Probably reflecting a form of anxiety about OH’s potentially incantatory dimension, the existence of these analyses also indicates the reflexivity of scientists who engage in OH: they too fear that OH is only a fad, a meaningless institutional injunction, a buzzword. They are working to ensure that this is not the case. This brings back to revisiting Chien’s proposition of OH as a concept whose imprecision is actually very productive. The vagueness of OH attracts heterogeneous actors and to resolve their possible conflicts. Staying in the blur, in the imprecision, is thus the condition on which these actors can continue to work together. When we look at things in the scientific literature on OH, we realize that researchers are not very comfortable with the blur, and they are working at getting out of the blur, out of the imprecision. Here, it is not the conceptual fuzziness itself that makes actors act, but rather the awareness of this fuzziness, and of the need to overcome it, by giving a more precise outline to OH. The search for precision becomes productive.

Thus, if OH’s power lies partly in being promoted by powerful actors, it is also based on its formal qualities (being a particular word), which encourages the mobilisation of the reflective and critical capacities of the individuals to whom it is addressed. The term “epistemic watchword” then seems to me more appropriate to describe a concept like OH which has the explicit ambition to lead to the production of knowledge and which has the (involuntary?) effect of encouraging its own collective elucidation, even its elicitation.
